# Morphological and Genetic Differentiation within the Southernmost Vector of Chagas Disease: *Triatoma patagonica* (Hemiptera – Reduviidae)

**DOI:** 10.1371/journal.pone.0168853

**Published:** 2016-12-22

**Authors:** Julieta Nattero, Sebastián Pita, Lucía Calleros, Liliana Crocco, Yanina Panzera, Claudia S. Rodríguez, Francisco Panzera

**Affiliations:** 1 Cátedra de Introducción a la Biología, Facultad de Ciencias Exactas Físicas y Naturales. Instituto de Investigaciones Biológicas y Tecnológicas -IIByT (CONICET—Universidad Nacional de Córdoba). Avda. Vélez Sarsfield, piso 2, Córdoba, Argentina; 2 Present address: Laboratorio de Eco-Epidemiología, Instituto de Ecología, Genética y Evolución de Buenos Aires, CONICET, EGE, FCEyN, UBA, Intendente Güiraldes 2160—Ciudad Universitaria—Pabellón 2, Buenos Aires, Argentina; 3 Sección Genética Evolutiva, Facultad de Ciencias, Universidad de la República. Iguá, Montevideo, Uruguay; Universidade Federal do Rio de Janeiro, BRAZIL

## Abstract

The epidemiological importance of Chagas disease vectors largely depends on their spreading ability and adaptation to domestic habitats. *Triatoma patagonica* is a secondary vector of Chagas disease endemic of Argentina, and it has been found colonizing domiciles and most commonly peridomiciliary structures in several Argentine provinces and morphological variation along its distribution range have been described. To asses if population differentiation represents geographic variants or true biological species, multiple genetic and phenotypic approaches and laboratory cross-breeding were performed in *T*. *patagonica* peridomestic populations. Analyses of chromatic variation of forewings, their size and the content of C-heterochromatin on chromosomes revealed that populations are structured following a North-South latitudinal variation. Cytochrome c oxidase I mitochondrial gene (COI) nucleotide analysis showed a mean genetic distance of 5.2% between the most distant populations. The cross-breeding experiments suggest a partial reproductive isolation between some populations with 40% of couples not laying eggs and low hatching efficiency. Our findings reveal phenotypic and genetic variations that suggest an incipient differentiation processes among *T*. *patagonica* populations with a pronounced phenotypic and genetic divergence between the most distant populations. The population differentiation here reported is probably related to differential environmental conditions and it could reflect the occurrence of an incipient speciation process in *T*. *patagonica*.

## Introduction

Chagas disease is a neglected tropical disease considered a serious threat to human health in Latin America that also represents an emerging health problem in the United States and Europe [1]. In the Americas, hemipteran insects of the Triatominae subfamily are vectors of *Trypanosoma cruzi*, etiologic agent of Chagas disease. This subfamily includes 150 species of obligatory hematophagous insects, classified in 5 tribes and 18 genera mainly distributed in the New World [2].

**Fig 1:**
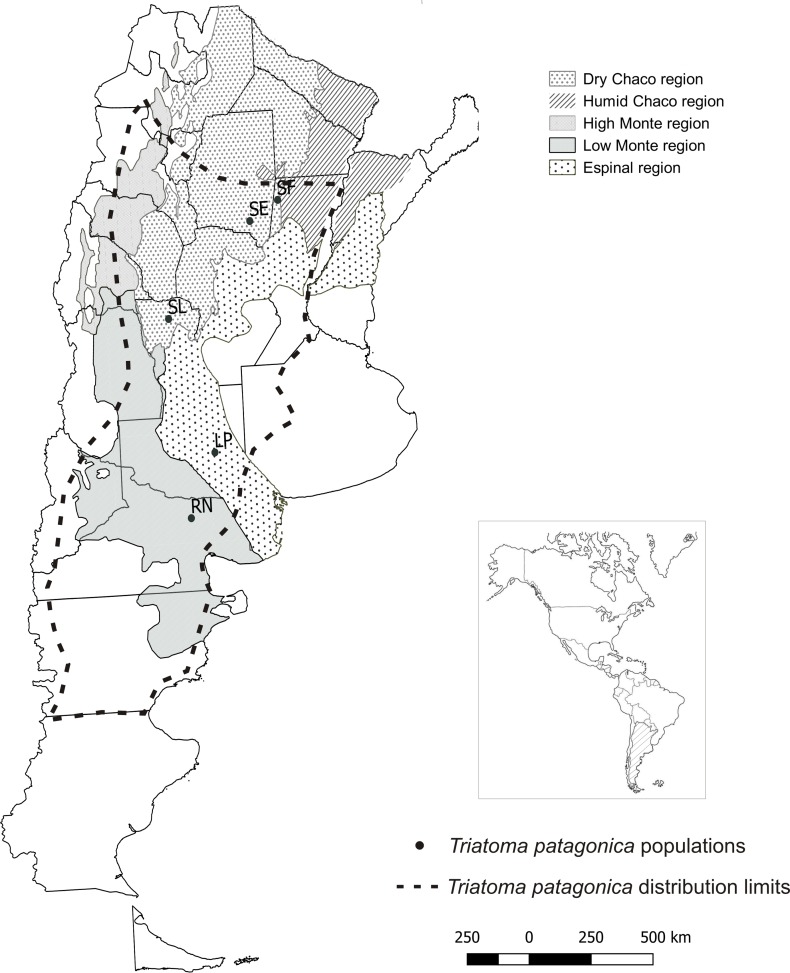
Geographic location of *Triatoma patagonica* populations here studied, including distribution limits of the species and ecological regions. SF = Santa Fe, SE = Santiago del Estero, SL = San Luis, LP = La Pampa, RN = Rio Negro. Figure is similar but not identical to the original image, and is therefore for illustrative purposes only.

The epidemiologic importance of Chagas disease vectors mainly depends on the spreading ability and adaptation to domestic habitats [3]. *Triatoma infestans* is the most successfully triatomine species adapted to domestic environments and the most important vector in the Southern cone of Latin America. Since 1991, large-scale insecticide spraying campaigns were successfully implemented in several countries for the elimination of domestic *T*. *infestans*. In some regions, when the primary vector is eliminated, other triatomine species become epidemiologically important. Included in these secondary vectors is *Triatoma patagonica*, an endemic species of Argentina with a wide geographical range from the north to the south of this country [4] (Fig 1). This species is mainly sylvatic but it has been found colonizing domiciles and peridomiciliary structures in several Argentine provinces [5,6]. Different authors have shown the species efficiency as a *Trypanosoma cruzi* vector [7,8].

Phenotypic and genetic studies are essential to known taxonomic status, population structure as well as the differentiation and dispersal processes. The interplay among gene flow, selection and genetic drift across biogeographic regions often results in a mosaic of phenotypically differentiated populations [9–11]. Factors that influence the degree of phenotypic divergence among populations can be affected by geographic factors [12,13]. Insect species are unable to internally regulate their body temperatures and are directly affected by temperature and climate variables related to thermoregulation. Temperature has long been considered the main environmental driver of geographic clines in body size in insects [14].

Triatominae species are considered plastic insects that respond rapidly to environmental changes, which might represent a common evolutionary route to new species [15]. In Triatominae, wing size and shape variations have been used as phenotypic markers for different objectives, such as the delimitation of cryptic species [16], spatial structure and origins of house re-infestation [17]. Constitutive heterochromatin revealed by C-banding is a successful chromosomal marker for detection of interspecific and intraspecific variations in triatomines from different genera [18]. Significant variations in C-heterochromatin content allowed us to distinguish cryptic species in the *Triatoma* genus [19, 20]. DNA-based methods, particularly mitochondrial genes, have proven to be effective tools for assessing population genetics, recognition of morphologically similar species and for inferring phylogenetic relationships in Triatominae [21–24].

Population studies on *T*. *patagonica* are very scarce. Different patterns on the corium and connexivum related to their geographical distribution and wet conditions have been described [4]. Also, variations in antennal phenotype have suggested phenotypic plasticity and a great adaptation capacity to a wide range of habitats [25]. Chromosomal information of this species is restricted to individuals from Santa Fe’s province, which has a diploid chromosome number of 22 chromosomes with C-heterochromatin regions in all autosomes and both (XY) sex chromosomes [19]. Sequence data on *T*. *patagonica* is restricted to two mitochondrial gene fragments (16S and 12S rDNA) [21,26].

In this paper multiple approaches were performed on some *T*. *patagonica* populations to provide an assessment of their phenotypic and genetic variation, with the aim of understanding if they represent geographic variants or true biological species. We tested the hypotheses that both phenotypic and genetic variation will be structured in a latitudinal variation, associated with environmental conditions of the different ecological regions (eco-regions). Moreover, experimental reciprocal crosses between most distant *T*. *patagonica* populations were carried out in order to evaluate their reproductive compatibility and genetic isolation.

## Materials and Methods

### Insects and collection sites

Specimens were originally collected from peridomestic structures in five Argentinean provinces, representing North (Santiago del Estero and Santa Fe provinces, SE and SF respectively), Center (San Luis—SL) and South (La Pampa and Río Negro provinces, LP and RN respectively) regions (Table 1 and Fig 1).

**Table 1:** Geographic location, ecological regions and number of individuals from *T*. *patagonica* studied by the different techniques applied in this study. COI = cytochrome c oxidase I mitochondrial gene, GM = geometric morphometrics, CS = crossbreeding.

Province	Locality	Laboratory generation	Population name	Region	Eco-region	Number of individuals studied by			
						C-banding	COI	GM	CS
Santa Fe	9 de Julio		SF	North	Chaco (dry)	5	3	9	0
Santiago del Estero	Mitre	3^rd^	SE	North	Chaco (dry)	5	3	9	28
San Luis	San Martín		SL	Center	Chaco (dry)	1	4	12	0
La Pampa	Utracan	3^rd^	LP	South	Espinal	6	2	8	28
Rio Negro	Avellaneda	3^rd^	RN	South	Monte	6	5	10	0

SF individuals were collected from goat corrals, usually made of piled thorny shrubs and vertical posts. SL, LP, RN and SE insects were collected from chicken coops but with differences in their building materials. In SL, chicken coops were built with wire mesh and wood sticks and thatched. In LP and RN, chicken coops were made of cairns of stones, while in SE they were built with walls made out of wood sticks or mud bricks. Insects were supplied by the “Centro de Referencia de Vectores (CeReVe)” from the National Health Ministry from Argentina. All specimen collections were carried out by technical personnel from the National Health Ministry from Argentina. In all cases, owners of the domiciles gave permission to collect specimens. *T*. *patagonica* is not an endangered or protected species. Insects analyzed came from natural populations (SF and SL) and 3 laboratory colonies (SE, LP and RN) with no more than 3 laboratory generations. The geographic distance between the most distant collection sites (SE and RN) is around 1,300 kms. The geographic localization and number of specimens studied from each collection site, as well as the techniques applied, are described in Table 1.

Collection sites of *T*. *patagonica* belong to 3 different ecological regions from Argentina (Table 1 and Fig 1). Each one is characterized by dissimilar vegetation and predominant species, diverse temperatures and precipitation regimens that determine different soil types [27, 28]. The Dry Chaco eco-region, including North and Center populations, is characterized by a warm subtropical continental weather, where annual rainfall were markedly in summer and varied between 500 and 700 mm. The Espinal eco-region (LP population) showed a template and dry weather with a hydric deficit, while the Monte eco-region (RN population) presented a temperate-arid weather with annual rainfall of about 100 mm (Fig 1) [28].

It has been proposed that *T*. *patagonica* is highly variable regarding the pattern elements on the corium and connexivum, considering a geographic-ecological basis: the darker elements seem to be more extensive and intensive in the east (humid regions), and become more reduced in the semiarid western region of Argentina [4]. We compared chromatic variation for the corium of the wings and connexivum elements across studied populations.

Most individuals were concurrently analyzed with all applied techniques (Table 1). Wings and legs from freshly killed adults were stored in 70% ethanol for morphometric and DNA analyses, respectively. Gonads (testes and ovaries) were removed and fixed in an ethanol-acetic acid mixture (3:1) and stored at -20°C for C-banding technique.

### Wing geometric morphometrics

**Fig 2:**
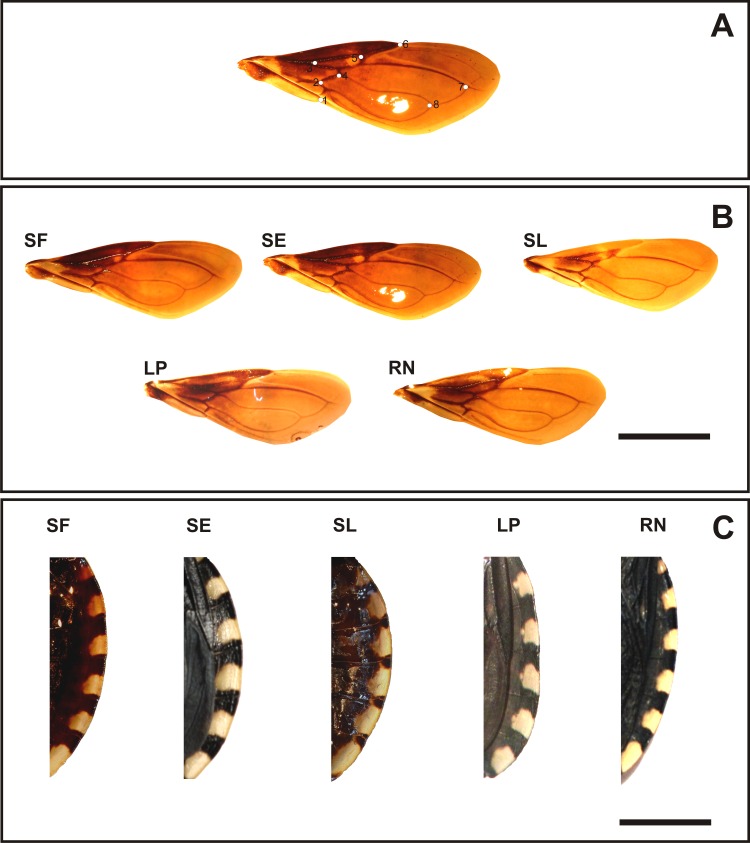
**(A): Forewing landmarks position used within venation intersections in *T*. *patagonica*. (B): Right forewings and (C) portion of the connexivum from individuals of the *T*. *patagonica* populations.** Populations named as in Table 1. Bar = 5 mm.

Forewings were mounted between microscope slides and cover slips [17]. Photographs of each right forewing were taken using a digital camera (Sony MVC-CD300, US) and a stereo-microscope (Zeiss SV11, Germany). Eight landmarks were identified in each wing along the outline and the venation intersections and the geometric coordinates of each landmark were digitalized using tpsDIG 2.17 [29] (Fig 2A). With these geometric coordinates, wing size and wing shape variation were obtained. Wing size was obtained with a single variable, the centroid size (CS). CS is defined as the square root of the sum of squared distances between the center of the configuration of landmarks and each individual landmark [30]. The CS was used to compare wing size among populations (S1 Table).

A generalized least squares Procrustes superimposition was used [30] for wing shape variation (S1 Table). To explore possible differences in wing shape among populations, we first performed a PCA to get orthogonal variables and then a CVA (on PCA scores, which are uncorrelated by construction) to see if forewing shape can distinguish the 5 populations. Statistics were performed using MorphoJ version 1.06f (http://www.flywings.org.uk/morphoj_page.htm). The relationship between CS and shape discrimination among populations (allometry) was estimated by the regression on size variation of the canonical axes derived from shape. We did not found allometric effects for any of the studied populations (results not shown).

### Cytogenetic studies

Air-dried chromosome preparations were made by squashing gonads in 50% acetic acid, freezing them in liquid nitrogen and removing the cover slip with a razor blade. Chromosome preparations were treated for C-banding to observe C-heterochromatin chromosomal distribution during mitosis and meiosis as previously described [19]. Microscope images were obtained under a Nikon Eclipse 80i epifluorescence microscope with a Nikon DS-5Mc-U2 digital cooled camera using Nikon Nis Elements 3.1 Advanced Research software and processed with Adobe Photoshop 8.0 software. For each individual, at least 30 meiotic metaphases I (MI) or II (MII) or diplotene stages were studied.

### DNA sequence analyses

For DNA extraction, one or two legs fixed in 70% ethanol were used from each specimen and total DNA was isolated by standard phenol-chloroform techniques. For each specimen, a 569-bp fragment of COI gene was amplified by PCR [23]. The PCR products were sent to Macrogen Inc. (Korea) for DNA sequencing. For all samples, sequencing was conducted in both forward and reverse directions. Sequences were assembled and manually curated by chromatogram evaluation using SeqMan software (DNASTAR). These sequences were evaluated by BLAST (http://www.blast.ncbi.nlm.nih.gov/Blast.cgi) to find the homologous sequences in GenBank. Sequences were deposited in the GenBank database (http://www.ncbi.nlm.nih.gov), under accession numbers KR139998 to KR140009 and KU842348 to KU842352. For phylogenetic analyses, we used as a triatomine species included in the same subcomplex (*T*. *guasayana*) [31] using COI sequences retrieved from GenBank as an out-group. Sequence alignment was performed using clustalW in MEGA6. Maximum likelihood (ML) phylogenetic analysis was implemented in MEGA6 [32] with a statistical support for tree topology obtained by bootstrapping [33] using 1000 iterations. MEGA6 was also used to find the best fitted substitution model. To build the ML topology, the nearest-neighbor-interchange (NNI) heuristic search method was used. Genetic nucleotide pairwise distances were calculated in MEGA6 using Kimura 2-parameter (K2-p), with the standard error derivate from 1000 bootstrap pseudo-replicates, and within-population nucleotide diversity (π) was calculated using DnaSP software. Tree images were obtained using Tree graph 2 software (http://treegraph.bioinfweb.info).

### Laboratory cross-breeding

Laboratory crosses were conducted in the Laboratory of insect vectors from the FCEFyN (UNC, Córdoba, Argentina) from fifth instar nymphs from SE and LP. Insects were kept in cylindrical glass bottles (500 cm^3^) covered with nylon mesh and containing vertically folded paper to allow insects’ access to food and to avoid humidity excess. During all experiments, insects were kept in the laboratory at 26 ± 2°C, 60–70% RH and a 12:12 h (L:D) photoperiod. Bugs were fed regularly every 15 days on pigeons (*Columba livia*). Once bugs had reached the adult stage, 28 crosses were made and each couple was placed in separate glass bottles. Once a week the number of eggs laid and eggs hatched were recorded. Crosses within each population were not performed since the number of fifth instar nymphs that CeReVe could supply was very limited. Nevertheless, intra-population crosses were performed with the same environmental conditions and feeding frequency for RN population and previously published [34].

## Results

### Morphometric studies

Three patterns of chromatic variation of forewings were observed across populations: dark pattern in North group (SF and SE populations), intermediate darkness pattern in South group (RN and LP) and a light pattern in Center group (SL individuals) (Fig 2B). Connexivum dark elements showed a similar pattern in the North and South populations and a light pattern in the Center population (Fig 2C). The pattern of the light elements of the connexivum is different across populations (Fig 2C).

**Fig 3:**
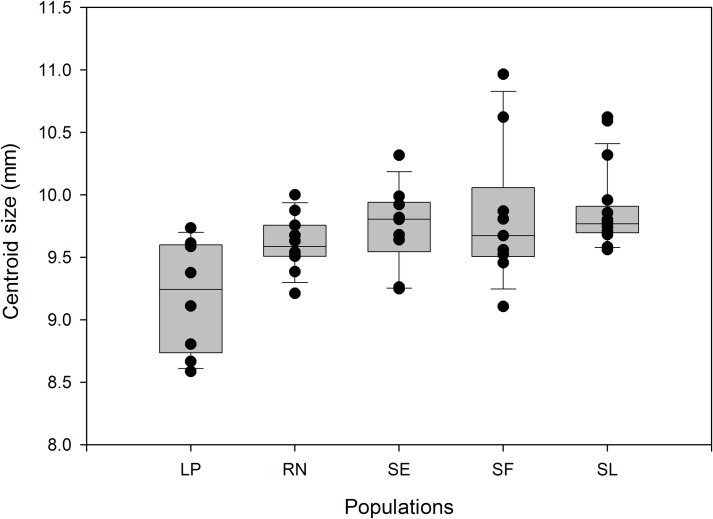
Box plots (median and standard deviation) of wing centroid size across the five studied *T*. *patagonica* populations. Populations named as in Table 1.

Comparisons of wing size between populations were shown in Fig 3. South group (LP and RN populations) showed smaller wings than in Center and North groups (SL, SF and SE populations) (Fig 3).

**Fig 4:**
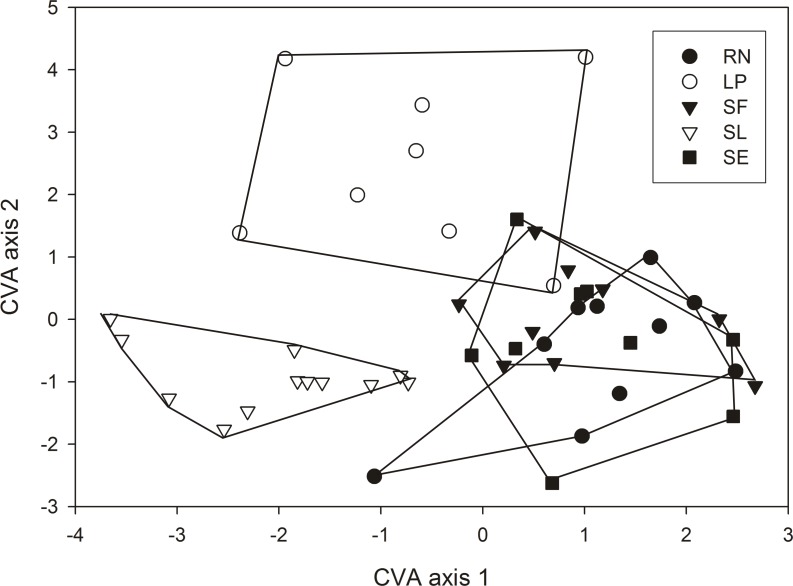
Factorial map in the plane of the two first axes from a canonical variate analysis of wing shape variation in five *T*. *patagonica* populations. For easy visualization, the lines connect the most external individuals of each population. Populations are named as in Table 1.

For wing shape analyses, the first two axes of the CVA performed with PCA scores, explained 37.11% and 27.15% of the variation, respectively. The factorial map distribution of each individual in the space of the first CVA axes showed that the SL and LP populations were highly differentiated in this space from the rest. RN, SE and SF individuals were overlapped (Fig 4).

### Cytogenetic studies

**Fig 5:**
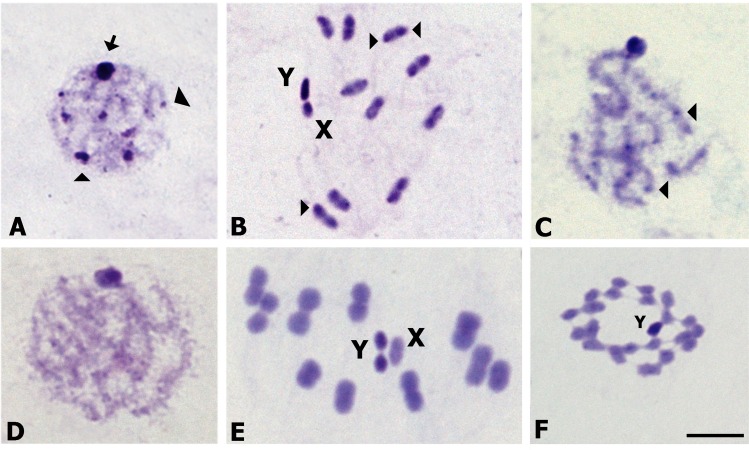
Meiosis and mitosis with C-banding of *T*. *patagonica*. All individuals have 22 chromosomes. North pattern: A and B. (A) Early meiotic prophase with several heterochromatic chromocenters spread in the nucleus (arrowheads). The large one is constituted by the associated sex chromosomes (arrow). (B) Second meiotic metaphase. The ten autosomal pairs presented C-heterochromatin in one or both chromosomal ends (arrowheads). The Y chromosome is entirely C-heterochromatic and the X chromosome presents a C-positive region and a C-negative one. Center pattern: (C) Early meiotic prophase with small C-dots in all autosomal bivalents (arrowheads). South pattern: D, E and F. Early meiotic prophase (D), first meiotic metaphase (E) and gonial prometaphase (F) without autosomal C-heterochromatin. Only the Y chromosome appears C-heterochromatic. Bar = 10 μm.

Male and female specimens from all populations analyzed showed the same diploid number (2n = 22) constituted by 20 autosomes plus two sex chromosomes (XY in males, XX in females). All individuals presented normal meiosis without meiotic irregularities. All specimens analyzed exhibit a C-heterochromatic Y chromosome and the autosomes may possess or lack C-heterochromatic regions. C-banding technique also revealed the existence of three C-heterochromatin distribution patterns. The North pattern includes all individuals of the North region (SE and SF populations). All autosomes showed large C-heterochromatic blocks in one or both chromosomal ends (Fig 5A and 5B), representing about 30% of the chromosome length. The X chromosome presents a C-positive region and a C-negative one (Fig 5B). During early meiotic prophase, the large size of C-blocks is clearly seen as heteropycnotic chromocenters (arrowheads). The large one is formed by the associated sex chromosomes (arrow Fig 5A). The Center pattern includes individuals from SL population (Center region). All autosomes presented a small C-dot in each chromosomal end, only observed during early meiotic prophase (Fig 5C). The X chromosome is euchromatic. The South pattern includes all individuals of the South region (LP and RN populations). All autosomes and X chromosomes do not have C-heterochromatin (Fig 5D–5F).

### DNA sequence analyses

**Fig 6:**
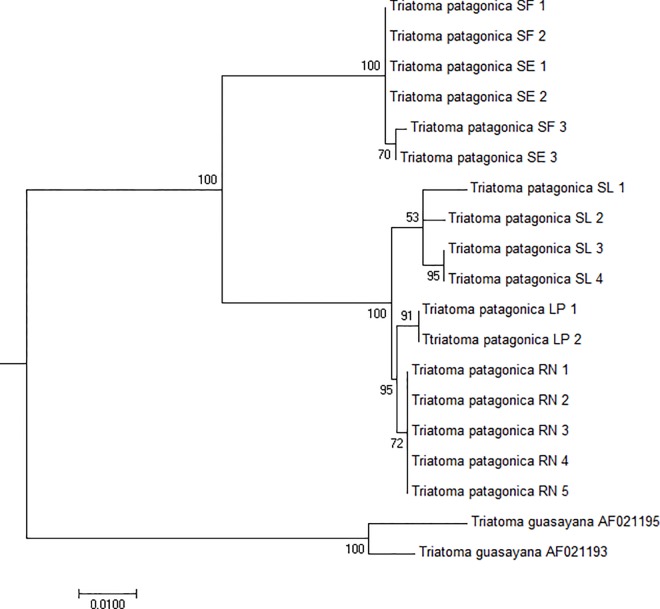
Maximum likelihood phylogenetic tree obtained with *T*. *patagonica* COI gene fragments. Tree topology reveals that *T*. *patagonica* is composed of two supported clades, represented by North (SE and SF individuals) and Center (SL individuals) plus South regions (LP and RN individuals). Numbers over nodes represent statistical support obtained through 1000 bootstrap replications. The numbers after the name of the species indicate the individual analyzed.

The 569 bp COI sequences analyses of 17 individuals showed 8 haplotypes, 3 of them represented by only one individual. Overall, there were 75 polymorphic sites, 17 of which were singletons. Maximum likelihood phylogenetic tree retrieved, using General Time Reverse (GTR) model, was well-supported containing two clades (100% bootstrap support): North clade including SF and SE individuals (3 haplotypes) and Center-South clade formed by SL (3 haplotypes) plus LP and RN individuals (2 haplotypes) (Fig 6).

Mean genetic distance (K2-p) between North and Center-South clades is 5.2 ± 0.9% (from 5.0 to 5.3 ± 0.9%). Genetic distance within Center-South clade (SL, LP and RN haplotypes) is from 0.5 to 1.3 ± 0.4%. All *T*. *patagonica* populations have genetic distances greater than 8.9% compared to *T*. *guasayana* used as out-group (Table 2).

**Table 2:** Genetic nucleotide pairwise distances (k2-p) between *T*. *patagonica* populations, using *T*. *guasayana* as an out-group. Distances are expressed in percentage numbers. Within-population nucleotide diversity (π) is expressed on the diagonal.

Populations	LP	SE	SL	SF	RN	OOUT-GROUP
LP	0.0					
SE	5.2±1.0	0.0012				
SL	1.3±0.4	5.3±0.9	0.0076			
SF	5.2±1.0	0.1±0.1	5.3±0.9	0.0023		
RN	0.5±0.3	5.0±0.9	1.2±0.4	5.0±0.9	0.0	
OUT-GROUP	9.9±1.4	8.9±1.2	9.3±1.3	8.9±1.2	9.7±1.4	0.23

### Cross-breeding experiments

Of the 28 experimental couples between North (SE) and South (LP) populations, 60% of them produced eggs, of which an average of 53% (37% and 67%) have hatched eggs (Table 3, S2 Table).

**Table 3:** Reproductive parameters for the cross-breeding experiments in *T*. *patagonica* individuals. Population named as in Table 1. *Data extracted from elsewhere [34].

Crosses	No. of couples	No. couples with laid eggs (%)	No. couples with hatched eggs (%)	No. eggs laid	No. eggs hatched
				Mean ± SD	Mean ± SD
SE ♀ x LP ♂	13	8/13 (61%)	3/8 (37%)	8.6 ± 10.69	14.00 ± 8.19
LP ♀ x SE ♂	15	9/15 (60%)	6/9 (67%)	15.0 ± 16.08	12.83 ± 8.82
RN ♀ x RN ♂*	15	15/15 (100%)	15/15 (100%)	44.15 ± 5.08	33.46 ± 10.07

The number of laid and hatched eggs per couple varied greatly between the crosses SE x LP and LP x SE with RN x RN (Table 3). These rates show quite low values compared to the ones obtained among individuals from RN population used as a control group (Table 3).

## Discussion

Speciation in Triatominae has been related to rapid morphological changes associated with ecological or environmental factors [15,35]. During the speciation process, variation degree in phenotypic and molecular traits, as well as the strength of reproductive isolation, may vary quantitatively [36]. *Triatoma patagonica* presents the southernmost distribution within all the Triatominae subfamily with broad North-South and East-West ranges that include a wide variety of environmental conditions, especially with regards to temperature and humidity [4]. Meteorological data from collection sites included in this study showed an extensive diversity with regards to geographic and climatic conditions. Chromatic variation of forewings and connexivum associated with humidity proposed by [4] does not agree with our results. Corium follows a North-South variation pattern, being darker in North populations. Connexivum variation is similar between North and South populations.

Wing size as well as the C-heterochromatin content showed latitudinal variation. It has been proposed that wing size variation may be strongly influenced by ecological attributes of populations, while wing shape is thought to be affected by genetic and historical attributes [35]. Wing size showed also eco-regions associated variation: individuals from Chaco eco-region (North and Centre populations) present bigger wings than those from the Monte or Espinal eco-regions (South populations). These three eco-regions varied in climatic conditions and also in the rainfalls amount and seasonality [27]. Temperature could influence body or other structures size during insect development [14]. In other Triatominae species, temperature influence eggs and nymphal development rates [37,38]. Development rate related to geographic or climatic variables may represent a selective pressure associated with the environment or a direct positive relation between wing development and temperature.

C-heterochromatin content differentiates three groups with a North-South decreasing C-content (Fig 5). Although C-heterochromatin polymorphism is frequently observed in several triatomine species [18], the pronounced autosomal differences observed among North (35% of C-content) and South (0%) individuals had not been described in conspecific populations. Variations of this magnitude have only been detected among sibling *Triatoma* species from North [39] and South America [19,20,31]. Extensive literature describes possible effects and adaptive functions of heterochromatin on both of plants and animals [40]. Changes in quantity and chromosomal position of C-heterochromatin affect the recombination level and expression of adjacent euchromatic regions (position effect variegation) [41]. In *T*. *infestans*, marked C-heterochromatin population differentiation could be reflecting adaptive genomic changes that contribute to the ability to survive and reproduce in different environments [41,42]. Heterochromatin variation in *T*. *patagonica* populations could also be related to adaptive changes associated with different environmental conditions.

For mitochondrial COI gene fragments, the biggest mean pairwise nucleotide distance (5.2%) was observed between North and Center-South *T*. *patagonica* clades (Table 2 and Fig 6). A similar value is reported between *T*. *sordida sensu stricto* and a new putative species *T*. *sordida Argentina* (5.3%) [20], but lower than reported to separate closely related triatomine species (7.1%) [23]. Considering that the persistence of gene flow among populations is unlikely, this nucleotide divergence might be due to several causes: a recent North-South dispersion process, and/or adaptive genetic changes associated with different ecological or environmental conditions.

Hybridization studies are powerful tools for determining the existence of isolating mechanisms that lead to population divergence and speciation [43]. Reproductive isolation is the best criteria to evaluate a population taxonomic status [44]. In triatomines, experimental crosses have been useful to make inferences on the genetic relationships, reproductive compatibilities and taxonomic status of populations or sibling taxa [45–48]. In this regard, our cross-breeding experiments suggest the existence of some degree of divergence between SE and LP populations, with 40% of couples not laying eggs, low number of eggs laid and low efficiency of eggs hatching (Table 3). Our cross breeding experiments have some limitations. First, the control groups do not include all cross populations. Second, we do not perform experimental crosses between geographically closest populations (e.g., SE x SF, SF x SL, or LP x RN). Third, our experimental design precludes the assessment of some pre-mating barriers, such as behavioral and pheromone. Also, in order to precisely determine the post-zygotic mechanisms acting among distant *T*. *patagonica* populations, it would be necessary to analyze hybrids fertility, including chromosomal behavior in F1 meiotic products.

Regardless of which processes are acting, the extreme chromosomal differentiation (C-heterochromatin amount), forewings corium and connexivum chromatic variation, wing size differences, COI gene sequences nucleotide differentiation and partial reproductive isolation determined by experimental crosses, suggest *T*. *patagonica* populations are undergoing an incipient speciation process. All these results suggest that *T*. *patagonica* should be considered as a highly polymorphic species with a pronounced phenotypic and genetic divergence between the most distant populations. The genetic and phenetic variations here reported are probably associated with broad eco-regional differentiation (dry Chaco, Monte and Espinal). Analyses on other populations not sampled in this study, such as humid Chaco, humid Pampa, and the Patagonian steppe, could help to clarify the mechanisms involved in the *T*. *patagonica* population differentiation.

## Supporting Information

S1 Table Procruste coordinates and centroide size of right forewings included in geometric morphometric study. (PDF)

S2 Table Number of matings, eggs laid and hatched from cross-breeding experiments in *T*. *patagonica* from SE and LP populations (PDF)
