# Supplementary material for: Morphological and Genetic Differentiation within the Southernmost Vector of Chagas Disease: Triatoma patagonica (Hemiptera – Reduviidae)
Source: PLoS One. 2016 Dec 22;11(12):e0168853. doi: 10.1371/journal.pone.0168853 (PMC5179239; doi:10.1371/journal.pone.0168853)
Supplement: S1 Table — (PDF) [file pone.0168853.s001.pdf]

Population	Centroid Size	ProcCoord1	ProcCoord2	ProcCoord3	ProcCoord4	ProcCoord5	ProcCoord6
SE	9.9887	-0.30402819	0.12698355	-0.31548786	0.03668966	-0.35363081	-0.06982231
SE	9.8038	-0.30553961	0.11969546	-0.30980926	0.03124297	-0.34612148	-0.06423148
SE	10.3154	-0.30522316	0.11992083	-0.30736717	0.02890404	-0.34333507	-0.06443463
SE	9.9219	-0.30841006	0.12889759	-0.30767634	0.0341755	-0.33094864	-0.06522656
SE	9.2478	-0.31340066	0.12972588	-0.3218226	0.03609266	-0.3306467	-0.07481467
SE	9.6791	-0.30574813	0.13618202	-0.31856005	0.03014318	-0.33237293	-0.06460062
SE	9.8187	-0.31098312	0.13014355	-0.29126652	0.06495648	-0.350242	-0.08254024
SE	9.6395	-0.30017153	0.1170483	-0.31272241	0.03257246	-0.34926753	-0.06502024
SE	9.2608	-0.30466399	0.12248593	-0.30843273	0.03148609	-0.34231902	-0.06672005
RN	9.3839	-0.30106735	0.13179583	-0.30781695	0.03439447	-0.35067622	-0.06836926
RN	9.9997	-0.30180997	0.13252727	-0.30643506	0.03567579	-0.36363488	-0.06681345
RN	9.8741	-0.30861519	0.12543886	-0.31243719	0.0297608	-0.34531249	-0.06518424
RN	9.5238	-0.29370963	0.12779525	-0.31285068	0.02747288	-0.34119467	-0.0661956
RN	9.6756	-0.31520523	0.12676645	-0.30874717	0.03312695	-0.32091409	-0.07259352
RN	9.2107	-0.31000778	0.13515778	-0.3159803	0.03226622	-0.35680898	-0.06634781
RN	9.5076	-0.2938284	0.11735811	-0.30185187	0.03053255	-0.37941473	-0.06238289
RN	9.5410	-0.3171644	0.12980943	-0.31396604	0.03062413	-0.33900263	-0.06643157
RN	9.7566	-0.30890896	0.13348455	-0.31447793	0.03205523	-0.33807359	-0.06166033
RN	9.6294	-0.30587562	0.12002957	-0.30426151	0.03029064	-0.35827657	-0.06387533
LP	9.5860	-0.31876749	0.12059811	-0.32306397	0.02660923	-0.32028935	-0.05709173
LP	9.1082	-0.30158559	0.13544472	-0.3003641	0.03415053	-0.33888525	-0.06584477
LP	9.7345	-0.31550077	0.11716082	-0.3177255	0.02729248	-0.33271795	-0.06036206
LP	9.3760	-0.32198028	0.12111843	-0.30809352	0.05829999	-0.32280782	-0.06887846
LP	9.6136	-0.31597356	0.12751535	-0.31516102	0.02823871	-0.32976394	-0.06158525
LP	8.5859	-0.31279365	0.11371755	-0.31356768	0.02895627	-0.34620769	-0.05901005
LP	8.6660	-0.32335955	0.12720671	-0.29933527	0.06668866	-0.32738061	-0.0749438
LP	8.8048	-0.31835167	0.13224232	-0.32451364	0.02852114	-0.31733837	-0.06156337
SF	10.6214	-0.30535963	0.11914053	-0.31009161	0.02918994	-0.35181979	-0.05958253
SF	10.9650	-0.29926727	0.12256657	-0.30578565	0.03151833	-0.34735765	-0.05666933
SF	9.6721	-0.28804545	0.1215385	-0.30138844	0.0348547	-0.37831355	-0.05849634
SF	9.8691	-0.30037727	0.13122092	-0.31102331	0.03293222	-0.33695234	-0.06346017
SF	9.5593	-0.3057417	0.11903073	-0.31012573	0.02921736	-0.33874689	-0.05758631
SF	9.4562	-0.29841224	0.12323683	-0.31044658	0.02842833	-0.33722273	-0.06589933
SF	9.5238	-0.29864153	0.12101217	-0.30855265	0.0291339	-0.32409786	-0.06446201
SF	9.8056	-0.29515505	0.11896989	-0.28714785	0.05566272	-0.35550503	-0.06871396
SF	9.1056	-0.30631614	0.13003307	-0.30893362	0.03834244	-0.34610456	-0.06789454
SL	10.6206	-0.29825649	0.12230636	-0.2966434	0.02693396	-0.36916831	-0.06107413
SL	9.7452	-0.3109144	0.12676608	-0.32101852	0.03346064	-0.33749458	-0.0659564
SL	9.5837	-0.30491278	0.12032427	-0.30708212	0.02716468	-0.35157017	-0.06521109
SL	10.3173	-0.30295845	0.12443619	-0.31054845	0.03401721	-0.34525801	-0.06227594
SL	9.9589	-0.31142956	0.12081715	-0.30728243	0.03284474	-0.34623808	-0.05836978
SL	9.7094	-0.31225616	0.12398984	-0.30815435	0.03039079	-0.34428996	-0.06193089
SL	9.5615	-0.3026854	0.12487345	-0.31478794	0.03122859	-0.33896659	-0.05510549
SL	9.8013	-0.31644585	0.12092934	-0.31442517	0.02894191	-0.33484056	-0.06676187
SL	9.7571	-0.30530742	0.12036639	-0.30331546	0.03165951	-0.34088909	-0.06028403
SL	9.8553	-0.29899311	0.12192698	-0.31363803	0.0294619	-0.32900697	-0.05976773

SL	9.6807	-0.29467657	0.12804098	-0.3139908	0.03020977	-0.33776366	-0.05745619
SL	9.7770	-0.30658307	0.12389146	-0.30925265	0.02790904	-0.34170662	-0.06233149

ProcCoord7	ProcCoord8	ProcCoord9	ProcCoord10	ProcCoord11	ProcCoord12	ProcCoord13	ProcCoord14
-0.20370643	-0.04770065	-0.09772926	-0.12189339	0.53482867	-0.04974151	0.46099696	0.00343329
-0.22382248	-0.04173216	-0.09467374	-0.12019123	0.53221705	-0.04892763	0.46574282	0.00311414
-0.2209381	-0.03927126	-0.09984933	-0.11452136	0.54777955	-0.05235467	0.46480643	0.00550177
-0.23728766	-0.03930816	-0.09505071	-0.12992245	0.54683015	-0.04314246	0.45236109	0.00768092
-0.21751831	-0.03057687	-0.09129525	-0.12271486	0.54501605	-0.03867598	0.4617193	-0.00442537
-0.22815953	-0.04913324	-0.09592971	-0.12901613	0.53367121	-0.04537961	0.45419307	0.00888362
-0.21413405	-0.05610769	-0.09805094	-0.1373146	0.54094268	-0.0552068	0.45849047	0.00634385
-0.22368288	-0.0441843	-0.1038367	-0.11390935	0.53650814	-0.03914624	0.4556272	0.00585758
-0.21941235	-0.03866064	-0.09225566	-0.12231333	0.54963139	-0.05750693	0.46203994	-0.00335207
-0.23476499	-0.04281307	-0.0721027	-0.13511913	0.52916134	-0.0567273	0.4609741	0.00629095
-0.22191045	-0.03938532	-0.06142144	-0.1284509	0.5487987	-0.05291619	0.46154408	0.00348585
-0.22729109	-0.03914968	-0.08182493	-0.12583297	0.5303799	-0.05630397	0.46025074	0.01165772
-0.23426416	-0.03798716	-0.09241754	-0.12703006	0.54829603	-0.05100251	0.4523432	0.00651933
-0.22452646	-0.03344496	-0.09352042	-0.12035103	0.56585108	-0.07209678	0.45504695	0.0094687
-0.2149927	-0.04089385	-0.06368638	-0.12919161	0.54600425	-0.05818279	0.44391444	0.01194864
-0.21301124	-0.03607219	-0.07467641	-0.11777293	0.55920793	-0.05588213	0.45122159	0.00865282
-0.22294241	-0.03655053	-0.07852735	-0.12850185	0.54197103	-0.05135791	0.4470545	0.00191075
-0.21055832	-0.04736179	-0.09545642	-0.12497614	0.56248873	-0.05440723	0.44634148	0.00892626
-0.22713451	-0.03474463	-0.06853641	-0.12252355	0.54882675	-0.06582203	0.45232088	0.01307948
-0.22905241	-0.03323612	-0.09453788	-0.12315532	0.54033904	-0.04856232	0.45740014	0.01363683
-0.23371736	-0.0428234	-0.10040659	-0.13649744	0.54719177	-0.04327251	0.45746178	0.00935582
-0.2336479	-0.03892932	-0.08729488	-0.11299487	0.532324	-0.04775325	0.45948328	0.00773634
-0.25096795	-0.04135965	-0.06385349	-0.1307784	0.55006685	-0.05247793	0.45369297	0.01289363
-0.2421258	-0.04155949	-0.07496424	-0.1213669	0.54193573	-0.05666952	0.44510399	0.01595471
-0.21789219	-0.03605973	-0.08636735	-0.11070009	0.55311276	-0.05414611	0.45306025	0.01026524
-0.23438877	-0.04898449	-0.08151136	-0.13724835	0.54867165	-0.05145693	0.45732106	0.01187156
-0.23931286	-0.04150684	-0.08086595	-0.12203463	0.53832301	-0.04262982	0.4556654	0.0039248
-0.23269037	-0.04117809	-0.08240456	-0.12483049	0.52412729	-0.04938155	0.46076291	0.01109035
-0.23245205	-0.0422493	-0.09215898	-0.13626024	0.53582944	-0.04450248	0.46007495	0.00287945
-0.22487638	-0.03869477	-0.07593258	-0.1426048	0.53553625	-0.04401333	0.45493002	0.00526874
-0.23479806	-0.04245916	-0.09343071	-0.13224881	0.54160532	-0.04475858	0.45990611	0.01005273
-0.22591251	-0.04059142	-0.09439886	-0.11956009	0.56011829	-0.0527626	0.44800069	0.00643255
-0.23422672	-0.05028609	-0.10014291	-0.11079966	0.54828439	-0.0583428	0.45103168	0.01095548
-0.23927493	-0.04215212	-0.10601178	-0.11774138	0.56211722	-0.05838535	0.45082333	0.00552092
-0.23776111	-0.05299397	-0.09411618	-0.13599017	0.54928573	-0.0541774	0.45345611	0.01209592
-0.22345121	-0.04228515	-0.07816745	-0.1335307	0.54487865	-0.06212307	0.4544238	0.01098064
-0.23023745	-0.03821106	-0.08253766	-0.12415503	0.5420131	-0.04097209	0.45182024	0.00432339
-0.2310968	-0.04411171	-0.06845034	-0.12101501	0.53328783	-0.05780505	0.45964126	0.00282975
-0.22043982	-0.04726494	-0.09876044	-0.10495697	0.53131656	-0.04734154	0.4745033	0.00339948
-0.22885919	-0.0453629	-0.08799207	-0.11737646	0.55662932	-0.03904115	0.44707116	-0.00159521
-0.230459	-0.04152034	-0.07848335	-0.12135842	0.53567991	-0.04525674	0.47052116	0.00144959
-0.21910775	-0.04374899	-0.0895692	-0.11494187	0.55173441	-0.05145386	0.45529832	0.00508003
-0.23460666	-0.04030381	-0.08331933	-0.12050586	0.55866757	-0.03530937	0.4580786	0.00314136
-0.22293652	-0.03657309	-0.08137689	-0.11085812	0.54185646	-0.06037063	0.47015541	0.00163579
-0.2280264	-0.04317658	-0.09645937	-0.11560921	0.55312	-0.0478536	0.46701299	0.00136978
-0.23481462	-0.04390728	-0.09366978	-0.11022483	0.57831114	-0.05599082	0.44536003	0.00715098

-0.24511653	-0.04158176	-0.0716041	-0.12903324	0.56003341	-0.05537668	0.45128893	0.00730225
-0.22307158	-0.03988094	-0.09877846	-0.11736037	0.54438003	-0.04066376	0.46559461	-5.16E-04

ProcCoord15	ProcCoord16
0.27875691	0.12205136
0.28200669	0.12102994
0.26412685	0.11625529
0.28018217	0.10684562
0.26794818	0.10538921
0.29290607	0.11292078
0.26524348	0.12972546
0.29754571	0.10678179
0.25541241	0.134581
0.27629277	0.13054749
0.24486902	0.11587694
0.28485026	0.11961349
0.27379744	0.12042788
0.24201534	0.1291242
0.27155744	0.11524343
0.25235314	0.11556664
0.2825773	0.12049754
0.25864501	0.11393944
0.26293697	0.12356585
0.28797192	0.10120132
0.27030534	0.10948704
0.29507972	0.10784986
0.26394324	0.10118239
0.29094885	0.10947238
0.27065554	0.10697692
0.25998285	0.10686664
0.28639408	0.10304639
0.29747575	0.11555185
0.28111721	0.12271701
0.27809013	0.12214729
0.27507026	0.10872085
0.26680671	0.11581977
0.28113509	0.12270725
0.26363819	0.12707386
0.26694338	0.12514696
0.26367052	0.12647732
0.28300998	0.1108486
0.27604554	0.12583169
0.27694548	0.11388609
0.27191569	0.10719827
0.26769136	0.1113938
0.26634469	0.11261495
0.25761976	0.09198115
0.25801312	0.12305667
0.25386474	0.11352773
0.24645133	0.11135079

0.25182932	0.11789489
0.26941774	0.10895255
