# Supplementary material for: Morphological and Genetic Differentiation within the Southernmost Vector of Chagas Disease: Triatoma patagonica (Hemiptera – Reduviidae)
Source: PLoS One. 2016 Dec 22;11(12):e0168853. doi: 10.1371/journal.pone.0168853 (PMC5179239; doi:10.1371/journal.pone.0168853)
Supplement: S2 Table — (PDF) [file pone.0168853.s002.pdf]

Cross-breedings LP X SE

Couple	Females LP	Males SE	Number of matings	Number of eggs laid
1	344	350	2	25
2	344	350	4	1
7	344	350	2	16
11	344	350	3	26
13	344	350	2	11
14	344	350	2	22
21	344	350	1	4
25	344	350	2	23
26	344	350	1	1
5	344	350	0	0
8	344	350	0	0
15	344	350	0	0
18	344	350	0	0
22	344	350	0	0
23	344	350	0	0
		Mean	1.266666667	8.6
		SD	1.279880947	10.68911329

-breedings SE SE LP

Couple	Females SE	Males LP	Number of matings	Number of eggs laid
3	350	344	2	41
6	350	344	3	28
10	350	344	1	3
12	350	344	4	36
16	350	344	1	14
17	350	344	3	34
19	350	344	2	28
20	350	344	1	11
4	350	344	0	0
9	350	344	0	0
24	350	344	0	0
27	350	344	0	0
28	350	344	0	0
		Mean	1.307692308	15
		SD	1.377474463	16.07793519

Number of eggs hatched
23
0
10
19
5
19
1
0
0
0
0
0
0
0
0
5.133333333
8.373996031

Number of eggs hatched
0
23
0
0
12
0
0
7
0
0
0
0
0
3.230769231
6.989919482
